# Circulating MiR-1290 as a potential diagnostic and disease monitoring biomarker of human gastrointestinal tumors

**DOI:** 10.1186/s12885-021-08729-0

**Published:** 2021-09-03

**Authors:** Liyi Xu, Yangke Cai, Xiao Chen, Yongliang Zhu, Jianting Cai

**Affiliations:** 1 Department of Gastroenterology, The Second Affiliated Hospital, School of Medicine, Zhejiang University, Hangzhou, 310009 Zhejiang, Province China; 2 Emergency Department, The Second Affiliated Hospital, School of Medicine, Zhejiang University, Hangzhou, Zhejiang Province China

**Keywords:** Gastrointestinal tumor, Circulating miRNA, miR-1290, Diagnosis, Surveillance, Biomarker

## Abstract

**Background:**

Gastrointestinal tumors are a leading cause of mortality worldwide. As shown in our previous study, miR-1290 is overexpressed in colorectal cancer (CRC) and promotes tumor progression. We therefore aimed to explore the potential of circulating miR-1290 as a biomarker for gastrointestinal cancer.

**Methods:**

A serum miRNA sequencing analysis was performed. Then, circulating miRNA detection technologies were established. The expression of miR-1290 was analyzed in gastrointestinal tumor cell lines and culture supernatants. Expression levels of circulating miR-1290 in clinical samples were examined. Associations between miR-1290 expression and clinicopathologic characteristics were analyzed. Xenograft models were generated to assess the fluctuation in serum miR-1290 levels during disease progression.

**Results:**

Through miRNA sequencing, we identified that miR-1290 was overexpressed in serum samples from patients with CRC. We confirmed that human gastrointestinal tumor cells express and secrete miR-1290. The circulating miR-1290 levels was up-regulated in patients with pancreatic cancer (PC) (*p* < 0.01), CRC (*p* < 0.05), and gastric cancer (GC) (*p* < 0.01). High miR-1290 expression levels were associated with tumor size, lymphatic invasion, vascular invasion, distant metastasis, tumor differentiation and AJCC stage in patients with PC and CRC. The area under the curve (AUC) was 0.8857 in patients with PC, with 60.9% sensitivity and 90.0% specificity. The AUC was 0.7852 in patients with CRC, with 42.0% sensitivity and 90.0% specificity. In patients with GC, the AUC was 0.6576, with 26.0% sensitivity and 90.0% specificity. The in vivo model verified that the circulating miR-1290 level was significantly increased after tumor formation and decreased after drug treatment.

**Conclusions:**

Our findings indicate that circulating miR-1290 is a potential biomarker for gastrointestinal cancer diagnosis and monitoring.

**Supplementary Information:**

The online version contains supplementary material available at 10.1186/s12885-021-08729-0.

## Background

Gastrointestinal tumors are the most common cancers worldwide [1, 2]. Colorectal cancer (CRC) and pancreatic cancer (PC) have the highest incidence and mortality rates among gastrointestinal tumors in the United States [1]. Compared to Western countries, China has a similar incidence of CRC but a significantly higher incidence of liver cancer and gastric cancer (GC). In 2018, gastrointestinal (stomach, liver, and esophagus) cancer-related deaths accounted for 36.4% of tumor-related deaths in China, while digestive cancer-related deaths comprised less than 5% of total cancer-related deaths in Western countries [3]. This difference may be related to the low early detection rate in China and the lack of uniformity of clinical treatment strategies in different regions. Therefore, population-based tumor screening can significantly increase the tumor detection rate, reduce tumor-related mortality, and improve patient prognosis. An effective screening method with high population coverage and compliance could have great clinical significance.

MicroRNAs (miRNAs) are non-coding, single-stranded, small RNAs of approximately 19–23 nucleotides, which widely exist in various organisms. Notably, miRNAs directly affect the cellular stability of messenger RNA (mRNA), thereby regulating gene expression at the posttranscriptional level and forming complex regulatory networks in cell proliferation, differentiation, apoptosis, homeostasis and stress response [4]. Extracellular/circulating miRNAs exist in various biological fluids, such as serum, plasma, saliva, urine, cerebrospinal fluid, and breast milk. They are delivered to target cells and act as autocrine, paracrine, and/or endocrine modifiers of cell activity [5]. Circulating miRNAs are potential diagnostic and prognostic biomarkers for various diseases [6].

An appropriate detection method is an essential step for liquid biopsy based on cell-free miRNA. The extraction efficiency, stability of internal controls and methodological accuracy all must be considered before clinical application [7]. Although numerous studies have been conducted to screen and identify circulating miRNAs as efficient biomarkers for specific types of tumors, the diagnostic value of a certain biomarker in different tumors must be evaluated, considering the broad spectrum of tumor markers.

As shown in our previous study, miR-1290 is overexpressed in clinical tumor tissues from patients with colon cancer and promotes tumor progression. However, we have not clearly determined whether miR-1290 can serve as a marker for early diagnosis and disease monitoring. In this study, miR-1290 expression in the serum from patients with gastrointestinal tumors was analyzed through miRNA sequencing, and a circulating miRNA detection method was built and evaluated. The diagnostic value of miR-1290 was verified for different tumors (including PC, CRC, and GC), and clinical characteristics of patients were collected to study the correlation between miR-1290 expression and clinicopathologic features. Meanwhile, a xenograft tumor model was established to explore the role of serum miR-1290 in disease surveillance. Therefore, we aimed to explore the potential of circulating miR-1290 to serve as a biomarker for gastrointestinal cancer.

## Methods

### Patients and clinical specimens

In this study, blood samples from 46 patients with PC, 50 patients with CRC, 50 patients with GC, and 50 healthy individuals were obtained at the Second Affiliated Hospital of Zhejiang University School of Medicine, Hangzhou, China, between January 2017 and December, 2019. Tumor tissues and adjacent non-tumor tissues were collected from ten patients with CRC. All patients with gastrointestinal tumors were enrolled at the initial diagnosis of tumors, and pathological diagnoses were subsequently confirmed.

### Sample processing

Blood samples were obtained by venipuncture. Serum samples were collected from whole blood in coagulation tubes, and plasma samples were processed from whole blood collected in an EDTA anticoagulation tubes (BD, New Jersey, USA). Blood samples were stored at 4 °C and processed within 4 h. Each sample was centrifuged at 1900 g (3000 rpm) at 4 °C for 10 min, and the supernatant was placed in a new centrifuge tube and centrifuged again at 16000 g at 4 °C for 10 min to remove the residual nucleic acid attached to the cell debris. Serum/plasma samples that were able to be examined on the same day were stored at 2–8 °C, and samples requiring long-term storage were stored at − 80 °C.

### miRNA sequencing

Three pairs of serum samples from CRC patients and healthy controls matched for clinical characteristics such as age, sex, and past history, were selected from our study group for miRNA sequencing. Sequencing was performed on Illumina Hiseq2000/2500 system (LC Science, USA) following the manufacturer’s protocol. Differentially expressed miRNAs based on normalized deep-sequencing counts were analyzed using Student’s t test. The screening criteria were a fold change > 2 and *p* < 0.01. The Venn diagram of miRNAs was generated through the online analysis tool Draw Venn Diagram (http://bioinformatics.psb.ugent.be/webtools/Venn/). The volcano map, heatmap and cluster analysis were conducted through online analysis tools (https://www.omicstudio.cn/tool).

### Target gene prediction, functional enrichment analysis, interaction network analysis and phylogenetic analysis

The target genes of miRNAs were predicted by the TargetScan and miRanda. The Database for Annotation, Visualization and Integrated Discovery (DAVID; https://david.ncifcrf.gov/) was a web-accessible functional annotation tool for GO and KEGG pathway enrichment analysis. False discovery rate (FDR) < 0.01 was considered as the threshold value. miRNA-mRNA interaction network analysis was realized by the Cytoscape (version 3.7.1), an open bioinformatics software. The phylogenetic tree was built with MEGA (version 7.0.20).

### Cell culture

The PC cell lines AsPC-1, BxPC-3, and SW1990, the CRC cell lines HT-29, HCT116, RKO, SW620, and SW480, and the GC cell lines SGC7901 and BGC823 were purchased from the Cell Bank of the Shanghai Branch of the Chinese Academy of Sciences. Primary CRC P4 cells were established in our laboratory from primary colorectal cancer tissues. AsPC-1, BxPC-3, SGC7901, and BGC823 cells were cultured in RPMI-1640 medium (Corning, New York, USA); SW1990, SW620, and SW480 cells were cultured in Leibovitz’s L-15 medium (Corning, New York, USA); and HT-29 and HCT116 cells were cultured in McCoy’s 5A medium (Corning, New York, USA). RKO cells were cultured in Minimum Essential Medium (MEM) (Corning, New York, USA). All cell culture media were supplemented with penicillin G (100 U/ml), streptomycin (100 μg/ml) and 10% fetal bovine serum (FBS) and grown at 37 °C with 5% CO2.

### Internal and exogenous controls

The internal and exogenous controls were hsa-miR-16-5p (*H. sapiens*; 5′-UAGCAGCACGUAAAUAUUGGCG-3′) and cel-miR-39 (*C. elegans*; 5′-UCACCGGGUGUAAAUCAGCUUG-3′) (RIBOBIO, Guangzhou, China), respectively. Then, 1 nmol of the cel-miR-39 standard was dissolved in 50 μl of nuclease-free water to obtain a 20 μM stock solution. The stock solution was diluted to 10 nM, and 5 μl of the 10 nM cel-miR-39 standard was added to each 200 μl volume of sample during circulating miRNA extraction.

### Isolation of cell-free miRNA and total RNA

Cell-free miRNA (including circulating miRNA and cell-free miRNA in the cell supernatant) was isolated from 200 μl of serum or plasma using five different commercially available extraction kits or reagents (Table S1) according to the manufacturer’s protocol. Total RNA was extracted from tissue samples and cultured cells using TRIzol reagent (Invitrogen, CA, USA).

### Reverse transcription

Total miRNA pools were used as the template for cDNA synthesis with the miRNA First Strand cDNA Synthesis Kit (Sangon Biotech, Shanghai, China) according to the manufacturer’s instructions. The following conditions were used: 16 °C for 30 min, 37 °C for 30 min, and 85 °C for 5 min. The reverse transcription primer for hsa-miR-1290 was 5′- GTCGTATCCAGTGCAGGGTCCGAGGTATTCGCACTGGATACGACTCCCTG-3′ (Sangon Biotech, Shanghai, China), the reverse transcription primer for hsa-miR-16-5p was 5′- GTCGTATCCAGTGCAGGGTCCGAGGTATTCGCACTGGATACGACCGCCAA-3′ (Sangon Biotech, Shanghai, China), the reverse transcription primer for cel-miR-39 was 5′-GTCGTATCCAGTGCAGGGTCCGAGGTATTCGCACTGGATACGACCAAGCTGA-3′ (Sangon Biotech, Shanghai, China), and the reverse transcription primer for U6 was 5′- CGCTTCACGAATTTGCGTGTCAT-3′ (Sangon Biotech, Shanghai, China).

### Quantitative real-time PCR

Quantitative real-time PCR analyses were performed on a CFX connect system (Bio-Rad Laboratories) with Taq PCR Mix (Sangon Biotech, Shanghai, China). The following cycling conditions were used: 95 °C for 150 s; 40 cycles of 95 °C for 15 s, 60 °C for 30 s, and 72 °C for 60 s; followed by 72 °C for 10 min. Three biological replicates were completed for all samples. The primers for hsa-miR-1290 were as follows: forward, 5′-GCGCGTGGATTTTTGGAT-3′; reverse, 5′-AGTGCAGGGTCCGAGGTATT-3′; and probe, 5′-FAM-CGCACTGGATACGACTCCCT-TAMRA-N-3′ (Sangon Biotech, Shanghai, China). The primers for hsa-miR-16-5p were as follows: forward, 5′- CGCGTAGCAGCACGTAAATA-3′; reverse, 5′- AGTGCAGGGTCCGAGGTATT − 3′; and probe, 5′-FAM-CGCACTGGATACGACCGCC-TAMRA-N-3′ (Sangon Biotech, Shanghai, China). The primers for cel-miR-39 were as follows: forward, 5′-GGCGTCACCGGGTGTAAA-3′; reverse, 5′- AGTGCAGGGTCCGAGGTATT-3′; and probe, 5′-FAM-CAGCTTGGTCGTATCCAGTGCG-TAMRA-N-3′ (Sangon Biotech, Shanghai, China). The primers for U6 were as follows: forward, 5′- GCTTCGGCAGCACATATACTAAAAT-3′; reverse, 5′- CGCTTCACGAATTTGCGTGTCAT-3′; and probe, 5′-FAM- CAGAGAAGATTAGCATGGCCCCTG-N-3′ (Sangon Biotech, Shanghai, China). Finally, the expression level of miR-1290 was analyzed using the 2^-ΔΔCt^ method, with hsa-miR-16-5p and cel-miR-39 serving as internal and exogenous controls, respectively.

### In vivo xenograft mouse model

Female BALB/c nude mice at 4–6 weeks of age and weighing 18–20 g were purchased from the Shanghai Experimental Animal Center of the Chinese Academy of Sciences. The Ethics Committee of the Second Affiliated Hospital of Zhejiang University School of Medicine approved the animal experiments. HT29 cells in the logarithmic growth phase were collected, adjusted to a density of 2.5 × 10^7^ cells/ml, and injected into the subcutaneous tissue of nude mice at a volume of 0.1 ml per mouse. Overall, 15 mice were randomly divided into three groups with 5 mice in per group (the control group, the tumorigenic group without 5-FU treatment, and the tumorigenic group with 5-FU treatment) and raised in a specific-pathogen-free (SPF) environment. Animals in the two experimental groups were inoculated with tumor cells. After 1 week, when the xenografted tumors had grown to a certain size (approximately 100 mm^3^), animals in one group were intraperitoneally injected with 5-FU (20 mg·kg^− 1^·d^− 1^) every 3 days, while animals in the other group were treated with the same amount of saline. The length and width of the tumors were measured every 3 days by a researcher who was unaware of the group allocation until the experiment was ended. The tumor volume (V) was calculated using the following formula: V = length/2 × width^2^. The mice were placed in a 10 L chamber with 99% CO_2_ (3 L/min) for 5–10 min, and vital signs (including breath, heartbeat, and muscular tension) were closely monitored until death was confirmed. Serum samples were collected, and circulating miR-1290 levels were verified by RT-qPCR.

### Hematoxylin and eosin (H&E) staining

Tissue specimens were incubated in 10% formaldehyde for 48 h and then stored in ethanol and embedded in paraffin. The tissue blocks were cut into 4-μm sections and stained with H&E (BASO). Slides were observed under a microscope (Olympus, Japan) and the following features were used to recognize tumor cells: architectural atypia, hyperchromasia, condensed and fractured chromatin, increased mitotic figures and nuclei migration to the edge.

### Statistical analysis

Descriptive statistical analyses were performed to summarize the clinical features of patients. Quantitative data are presented as the mean ± standard deviation. The chi-square test was used to analyze the associations between miR-1290 expression and clinicopathological characteristics. Differences between two independent groups were assessed using Student’s t test. Comparisons among multiple groups were conducted using one-way analysis of variance (ANOVA). The sample size was calculated using the PASS software (version 11.0.7). The charting and statistical analyses were performed using GraphPad Prism (version 8.0.2) and SPSS (version 23.0) software. A *p* value of 0.05 or less was defined as statistically significant (*, *p* < 0.05, **, *p* < 0.01, and ***, *p* < 0.001).

## Results

### Up-regulation of miR-1290 in tumor tissues and serum from patients with CRC

**Fig. 1:**
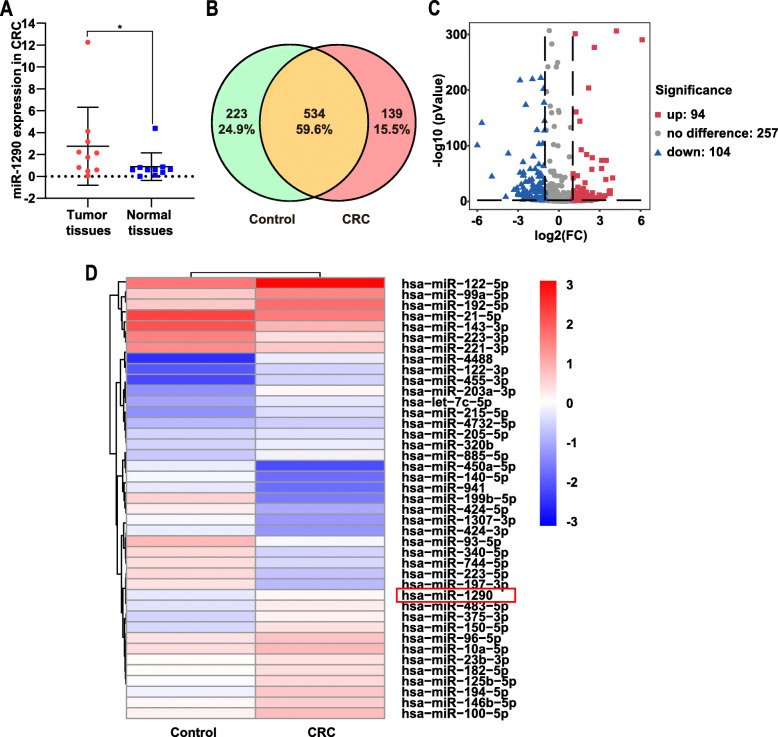
miR-1290 was upregulated in the tumor tissue and serum of patients with CRC. **a** Expression of miR-1290 in tumor tissues and paired normal tissues (*n* = 10). miR-1290 was significantly overexpressed in tumor tissues (*p* < 0.05). **b** Venn diagram of the commonly and differentially expressed miRNAs in different groups. 223 (24.9%) miRNAs were only detected in the control group, 139 (15.5%) miRNAs were only detected in the CRC group, and 534 (59.6%) miRNAs were detected in both groups. **c** Volcano plot of differentially expressed miRNAs. Two hundred sixty-one miRNAs were up-regulated and 395 miRNAs were down-regulated in serum samples from patients with CRC. (*p* < 0.01, |log2FC| > 1). **c** Heatmaps of cluster analysis for differentially expressed miRNAs with high expression levels (*p* < 0.01, |log2FC| > 1). Blue color represents a lower expression level and red color represent a higher expression level. * *p* < 0.05

Our previous study confirmed a substantial increase in miR-1290 expression in tumor tissues from patients with CRC and demonstrated that the up-regulation of miR-1290 impaired cytokinesis and affected the reprogramming of colon cancer cells. Therefore, miR-1290 plays an important role in CRC progression. We collected 10 pairs of tumor tissues and adjacent tissues to further verify the expression level of miR-1290 in CRC and found that miR-1290 was significantly overexpressed in tumor tissues (Fig. 1a).

Three pairs of serum samples from patients with CRC and controls matched for clinical characteristics, such as age, sex, and the past history, were selected for the miRNA sequencing analysis. We took the Venn intersection of the commonly and differentially expressed miRNAs in different groups. As shown in Fig. 1b, 223 (24.9%) miRNAs were only detected in the control group, 139 (15.5%) miRNAs were only detected in the CRC group, and 534 (59.6%) miRNAs were detected in both groups. In total, 656 differentially expressed miRNAs were detected in serum samples from patients with CRC, compared with samples from healthy controls; among them, 261 miRNAs were up-regulated and 395 miRNAs were down-regulated (*p* < 0.01). The miRNAs with very low expression levels were excluded, and a volcano graph analysis was performed. Compared with the control group, 94 miRNAs were up-regulated and 104 miRNAs were down-regulated in patients with CRC (|log2FC| > 1, *p* < 0.01) (Fig. 1c). Based on the miRNA expression profiles, differentially expressed miRNAs with high expression levels were chosen for cluster analysis. As shown in the heatmap, hsa-miR-1290 was significantly overexpressed in serum from patients with CRC (Fig. 1d), suggesting that miR-1290 might be a potential biomarker for gastrointestinal tumors.

In addition, 16 CRC-associated miRNAs were identified (FC > 3), as listed in Table S1. The potential target genes for these miRNAs were predicted using TargetScan and MiRanda, and 2646 target genes were finally predicted. To further understand the biological function of the identified target genes, GO and KEGG pathway enrichment analysis was performed by DAVID. As shown in Figure S1A-B, the Biological Process (BP) of target genes mainly focused on the Golgi vesicle transport, dephosphorylation, and protein localization to cell periphery. The Cellular Component (CC) of target genes were concentrated in synaptic membrane, cell-substrate adherens junction, and trans-Golgi network. The Molecular Function (MF) of target genes mainly enriched in phosphatase activity, phosphoric ester hydrolase activity, and protein Serine/Threonine kinase activity. Besides, the propanoate metabolism was the most important item in KEGG pathway analysis. To explore the evolutionary conservation on these biomarkers, the phylogenetic tree was plotted using the p distance and the average method. As shown in Figure S1C, these miRNAs were significantly separated into three branches. Besides, to further explore the miRNA interaction, we established a miRNA-mRNA regulatory network with 16 miRNAs and 61 predicted target genes, as depicted in Figure S2.

### Establishment and evaluation of circulating miRNA detection technologies for clinical Identification

A reliable methodology was needed to analyze the circulating miR-1290 expression levels in clinical samples. First, we used different methods to extract circulating miRNAs, including three extraction kits from QIAGEN and two TRIzol reagents from Invitrogen, each with different extraction principles, as shown in Table S2. We examined the serum miR-1290 levels in 20 healthy individuals using cel-miR-39 or miR-16-5p as a reference miRNA (Table S3). We obtained different results when choosing different reference miRNAs, indicating the importance of an appropriate control. The extraction efficiency of the miRNeasy Serum/Plasma Kit, miRNeasy Serum/Plasma Advanced Kit and TRIzol LS was better than that of the other two methods. Because the miRNeasy Serum/Plasma Kit and miRNeasy Serum/Plasma Advanced Kit are manufactured by QIAGEN and the miRNeasy Serum/Plasma Advanced Kit produced better results than the miRNeasy Serum/Plasma Kit, we further compared the extraction efficiency of the miRNeasy Serum/Plasma Advanced Kit and TRIzol LS for cell-free miRNAs.

**Fig. 2:**
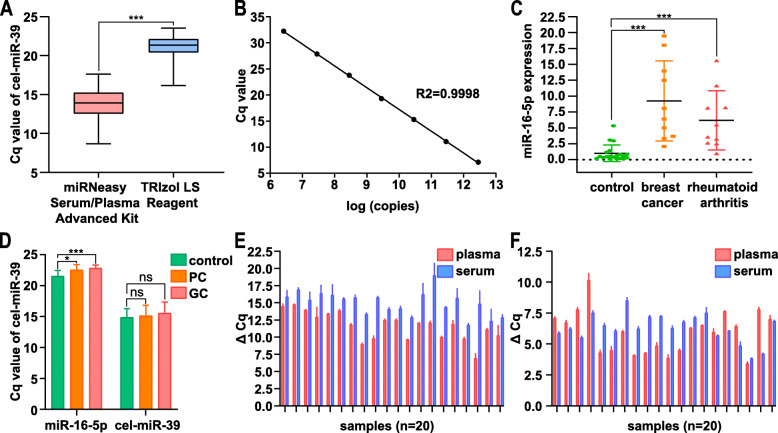
The establishment and evaluation of circulating miRNA detection methodology. **a** Extraction efficiency was compared between the miRNeasy Serum/Plasma Advanced Kit and TRIzol LS by RT-qPCR. **b** The standard curve of miR-1290 standards with different concentrations (100 nM, 10 nM, 1 nM, 10pM, 1pM, 0.1pM), y = −4.173x + 58.96, *R*^2^ = 0.9998. **c** Expression levels of circulating miR-16-5p were analyzed in breast cancer (*n* = 10) and rheumatoid arthritis (*n* = 10) by RT-qPCR. **d** Analysis of expression levels of endogenous and exogenous controls in PC, GC and control group by RT-qPCR. **d** Expression levels of miR-1290 were assessed in the serum and plasma of the same individuals by RT-qPCR, with miR-16-5p as an endogenous control. **e** Expression levels of miR-1290 were assessed in the serum and plasma of the same individuals by RT-qPCR, with cel-miR-39 as an exogenous control. * *p* < 0.05, *** *p* < 0.001, ns: no significance

The influence of the reference miRNA was eliminated by adding the same amount of cel-miR-39 (10 μM, 5 μl) to the same volume of serum. For the same amount of circulating cel-miR-39, the extraction efficiency of the miRNeasy Serum/Plasma Advanced Kit was apparently higher than that of TRIzol LS (Fig. 2a, *p* < 0.0001). Therefore, the miRNeasy Serum/Plasma Advanced Kit was chosen as the main method for subsequent experiments.

We tested miR-1290 standards at different concentrations (100 nM, 10 nM, 1 nM, 100 pM, 10 pM, 1 pM, 0.1 pM) and drew a standard curve (y = − 4.173x + 58.96, *R*^2^ = 0.9998) to further evaluate the recovery efficiency of the entire circulating miRNA detection system (Fig. 2b). Then, standard solutions of low, middle, and high concentrations were added to the sample solutions and analyzed in the same manner. The miRNA concentration in the sample was calculated from the standard curve, and the recovery rate was evaluated. The recovery efficiency in the three groups with a low, middle, and high standard concentration was 103, 116, and 87.8%, respectively (Table S4), which was within the acceptable range. The effect of interfering factors in the serum was also evaluated. We collected three samples with a high bilirubin, triglycerides, or rheumatoid factor level, and analyzed the recovery efficiency as described above. The recovery efficiency in the three groups was 119, 93.0, and 107%, respectively (Table S4), which was within the acceptable range of error. Therefore, the serum level of bilirubin, triglycerides, and rheumatoid factor did not interfere with the miRNA detection method.

Notably, miR-16-5p is a commonly used internal control for circulating miRNA detection. Previous studies have shown that miR-16-5p was overexpressed in breast cancer, chronic lymphocytic leukemia, rheumatoid arthritis, and other diseases. We collected serum samples from patients with different diseases, and evaluated miR-16-5p expression using cel-miR-39 as an exogenous control to verify the reliability of miR-16-5p as an internal control (Fig. 2c). The serum level of miR-16-5p in patients with breast cancer and patients with rheumatoid arthritis was significantly higher than that in healthy controls (*p* < 0.001). We also analyzed the Cq value of miR-16-5p and cel-miR-39 in patients with PC, patients with GC, and healthy controls. The results were shown in Fig. 2d. The Cq value of miR-16-5p in patients with PC and GC was much higher than that in healthy controls, while no significant difference in the Cq value of cel-miR-39 was observed, indicating that miR-16-5p expression fluctuates in gastrointestinal tumors and is not suitable for use as an internal control.

In the final part of the methodological evaluation, we collected plasma and serum samples from 20 healthy individuals, to evaluate the difference in miR-1290 expression between serum and plasma samples from the same individual (Fig. 2e-f). We observed higher miR-1290 expression in plasma than in serum when cel-miR-39 was used as the reference miRNA. In contrast, in some individuals, the expression of miR-1290 was higher in serum than in plasma when miR-16-5p was used as the reference miRNA, which also indicated that miR-16-5p was not a reliable internal control.

### Human gastrointestinal tumor cells express and secrete miR-1290

**Fig. 3:**
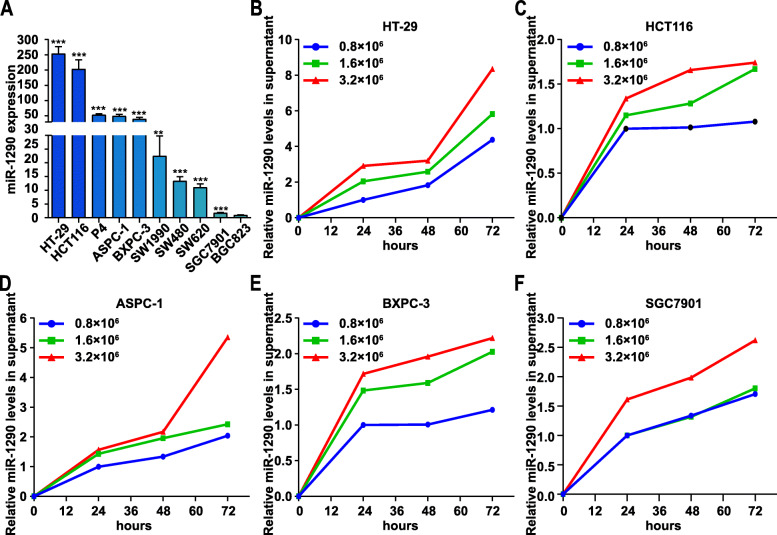
Human gastrointestinal tumor cells express and secret miR-1290. **a** Expression levels of miR-1290 were analyzed in different gastrointestinal cancer cells by RT-qPCR, including HT-29, HCT116, P4, ASPC-1, BXPC-3, SW1990, SW480, SW620, SGC7901, and BGC823. miR-1290 expression in HCT116, HT29, P4, ASPC-1 and BXPC-3 were relatively high. **b** miR-1290 expression levels in cellular supernatant of HT-29 when cultured with different cell densities for 24 h, 48 h, and 72 h. **c** miR-1290 expression levels in cellular supernatant of HCT116 when cultured with different cell densities for 24 h, 48 h, and 72 h. **d** miR-1290 expression levels in cellular supernatant of ASPC-1 when cultured with different cell densities for 24 h, 48 h, and 72 h. **e** miR-1290 expression levels in cellular supernatant of BXPC-3 when cultured with different cell densities for 24 h, 48 h, and 72 h. **f** miR-1290 expression levels in cellular supernatant of SGC7901 when cultured with different cell densities for 24 h, 48 h, and 72 h. ** *p* < 0.01, *** *p* < 0.001

We examined the expression of miR-1290 in human gastrointestinal tumor cell lines to clarify the source of circulating miR-1290. As shown in Fig. 3a, five CRC cell lines including HT29, HCT116, P4, SW480, and SW620, three PC cell lines including ASPC-1, BXPC-3, and SW1990, and two GC cell lines including SGC7901 and BGC823 were analyzed. The expression level of miR-1290 was lower in GC cell lines than in CRC and PC cell lines. The lowest miR-1290 expression level was observed in BGC823 cells, while miR-1290 was expressed at relatively high levels in HT29, HCT116, P4, ASPC-1, and BXPC-3 cells. We further examined miR-1290 expression in the culture supernatant of the five cell lines (HT-29, HCT116, ASPC-1, BXPC-3, and SGC7901) when cultured at different cell densities for 24, 48, and 72 h. As shown in Fig. 3b-f, gastrointestinal tumor cells expressed and secreted miR-1290 into the culture supernatant, and the miR-1290 expression level in the culture supernatant increased with both cell density and culture time. Therefore, circulating miR-1290 might be derived from tumor cells and secreted as a potential tumor biomarker.

### Circulating miR-1290 serves as a diagnostic biomarker for gastrointestinal tumors

In the subsequent validation phase, the circulating miR-1290 expression level was detected in 46 patients with PC, 50 patients with CRC, 50 patients with GC, and 50 healthy controls. The clinical characteristics of the participants were summarized in Table 1. Significant differences in sex, BMI, smoking history, or alcohol consumption were not observed between the PC and the control group. Patients in the PC group were older (*p* < 0.05) and a higher proportion of patients in this group had hypertension (*p* < 0.05) and diabetes (*p* < 0.05). Furthermore, the CEA (*p* < 0.001), CA199 (*p* < 0.001), CA125 (*p* < 0.001), CA242 (*p* < 0.001), and CA211 (*p* < 0.01) levels in the PC group were significantly higher than those in the control group. No significant variations in age, sex, BMI, alcohol consumption or diabetes history were observed between the CRC and the control group, but the ratio of smokers (*p* < 0.05) and patients with hypertension (*p* < 0.05) was much higher in the CRC group than in the control group. The CEA level (*p* < 0.001) in the CRC group was apparently higher than that in the control group, while the expression levels of the other biomarkers were similar in the CRC and the control group. In addition, no significant differences in age, sex, BMI, smoking history, alcohol consumption, hypertension history, or diabetes history were observed between the GC and the control group. CEA expression was higher in the GC group (*p* < 0.01) while the remaining biomarkers showed no significant difference in expression between the GC and the control group.

**Table 1:** Clinical characteristics of patients included in the study

Groups	Control (*n* = 50)	Group 1 (*n* = 46)		Group 2 (*n* = 50)		Group 3 (*n* = 50)	
		Pancreatic cancer	*p* value	Colorectal cancer	*p* value	Gastric cancer	*p* value
Age (year)			0.016*		0.102		0.151
< 65 y	34	20		26		27	
≥ 65 y	16	26		24		23	
Sex			0.084		0.105		0.841
Male	25	31		33		26	
Female	25	15		17		24	
BMI (kg/m^2^)			0.524		0.832		0.663
< 25	34	34		33		36	
≥ 25	16	12		17		14	
Smoke			0.167		0.021*		0.817
Yes	12	17		23		13	
No	38	29		27		37	
Alcohol			0.478		0.817		1.000
Yes	12	14		13		12	
No	38	32		37		38	
Hypertension			0.013*		0.047*		0.727
Yes	10	20		19		12	
No	40	26		31		38	
Diabetes			0.022*		1.000		0.629
Yes	5	13		5		4	
No	45	33		45		46	
CEA (ng/ml)			< 0.0001***		< 0.0001***		< 0.006**
< 5	50	30		33		43	
≥ 5	0	16		17		7	
CA199 (U/ml)			< 0.0001***		0.617		< 0.617
< 37	39	6		41		41	
≥ 37	11	40		9		9	
CA125 (U/ml)			< 0.0001***		0.169		< 0.137
< 35	46	24		49		41	
≥ 35	4	22		1		9	
CA242 (U/ml)			< 0.0001***		0.558		< 0.169
< 20	49	16		48		46	
≥ 20	1	30		2		4	
CA211 (ng/ml)			0.001**		0.307		0.092
< 5	49	34		47		45	
≥ 5	1	12		3		5	

* *p* < 0.05, ** *p* < 0.01, *** *p* < 0.001

**Fig. 4:**
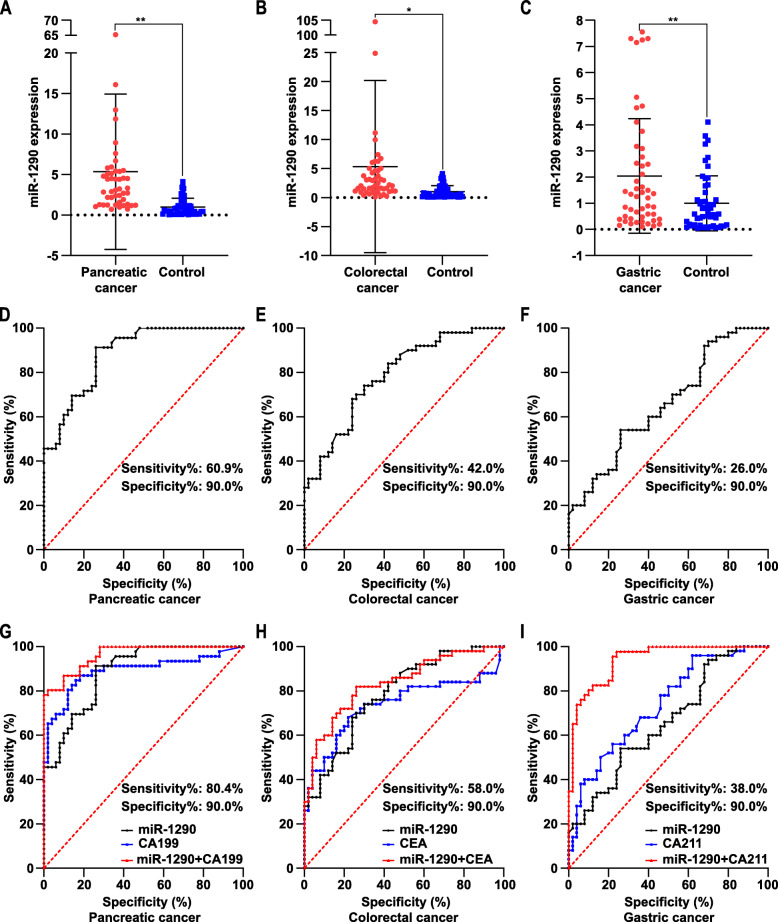
Circulating miR-1290 is a potential diagnostic biomarker in gastrointestinal tumors. **a** Serum miR-1290 expression levels in PC patients (*n* = 46) and healthy controls (*n* = 50) were analyzed by RT-qPCR. **b** Serum miR-1290 expression levels in CRC patients (*n* = 50) and healthy controls (*n* = 50) were analyzed by RT-qPCR. **c** serum miR-1290 expression levels in GC patients (*n* = 50) and healthy controls (*n* = 50) were analyzed by RT-qPCR. **d** Circulating miR-1290 yielded AUC value of 0.8857 with 67.4% sensitivity and 90.0% specificity in distinguishing PC patients from healthy controls (*p* < 0.001). **e** Circulating miR-1290 yielded AUC value of 0.7852 with 48.0% sensitivity and 90.0% specificity in distinguishing CRC patients from healthy controls (*p* < 0.001). **f** Circulating miR-1290 yielded AUC value of 0.6576 with 34.0% sensitivity and 90.0% specificity in distinguishing GC patients from healthy controls (*p* < 0.001). **g** Sensitivity and specificity of circulating miR-1290 combined with CA199 were 80.4 and 90.0% with the AUC value of 0.9626 in PC (*p* < 0.0001). **h** Sensitivity and specificity of circulating miR-1290 combined with CEA were 58.0 and 90.0% with the AUC value of 0.8348 in CRC (*p* < 0.0001). **i** Sensitivity and specificity of circulating miR-1290 combined with CA211 were 38.0 and 90.0% with the AUC value of 0.7788 in PC (*p* < 0.0001). * *p* < 0.05, ** *p* < 0.01

As shown in Fig. 4a-c, the expression level of circulating miR-1290 considerably increased in patients with PC (*p* < 0.01), CRC (*p* < 0.05), and GC (*p* < 0.01). Correlations between the serum miR-1290 expression level and clinicopathological characteristics of patients with gastrointestinal cancer were explored. In patients with PC (Table 2), significant correlations were not observed between the circulating miR-1290 level and age, sex, BMI, smoking history, alcohol consumption, hypertension history, diabetes history, or tumor site. High miR-1290 expression was associated with tumor size (*p* < 0.01), lymphatic invasion (*p* < 0.01), vascular invasion (*p* < 0.05), distant metastasis (*p* < 0.05), tumor differentiation (*p* < 0.05), and tumor AJCC stage (*p* < 0.05). However, no significant correlations were observed between the circulating miR-1290 expression level and the levels of other tumor biomarkers, including CEA, CA199, CA125, CA242, and CA211. In patients with CRC (Table 3), no clear associations were identified between the circulating miR-1290 expression levels and age, sex, BMI, smoking history, alcohol consumption, hypertension history, diabetes history, or tumor site. Significantly higher circulating miR-1290 levels were detected in CRC patients with larger tumors (*p* < 0.05), lymphatic invasion (*p* < 0.05), vascular invasion (*p* < 0.05), distant metastasis (*p* < 0.05), poorer tumor differentiation (*p* < 0.05), and more advanced AJCC stages (*p* < 0.05). The miR-1290 expression level was related to the CEA level (*p* < 0.05), while a significant correlation was not observed with the level of CA199, CA125, CA242, or CA211. In patients with GC (Table 4), significant correlations between the circulating miR-1290 expression level and age, sex, BMI, smoking, drinking, diabetes, or tumor location were not observed. The tumor size, lymphoid invasion, vascular invasion, distant metastasis, tumor differentiation degree, and the AJCC stage, as well as the levels of common tumor markers, also showed no remarkable correlation with circulating miR-1290 expression.

**Table 2:** Association between circulating miR-1290 expression and clinicopathologic characteristics in 46 pancreatic cancer patients

Variable	Total (*n* = 46)	miR-1290 expression		*p* value
		Low (*n* = 13)	High (*n* = 33)	
Age (year)	65.48 ± 1.77	69.15 ± 3.53	64.03 ± 2.01	0.195
Sex				0.975
Male	32	9	23	
Female	14	4	10	
BMI (kg/m^2^)	22.01 (19.69–25.40)	23.26 (20.00–26.88)	21.97 (19.16–25.13)	0.518
Smoke				0.057
Yes	17	2	15	
No	29	11	18	
Alcohol				0.164
Yes	14	2	12	
No	32	11	21	
Hypertension				0.818
Yes	20	6	14	
No	26	7	19	
Diabetes				0.813
Yes	13	4	9	
No	33	9	24	
Location				0.894
Head	29	8	21	
Body and tail	17	5	12	
Tumor size (cm)	3.55 (2.32–4.53)	2.30 (1.80–2.80)	4.00 (2.87–4.60)	0.002**
Lymphatic invasion				0.003**
Positive	35	6	29	
Negative	11	7	4	
Venous invasion				0.030*
Positive	32	6	26	
Negative	14	7	7	
Distant metastasis				0.022*
Positive	23	3	20	
Negative	23	10	13	
Differentiation				0.036*
Well	7	3	4	
Moderate	22	9	13	
Poor	17	1	16	
TNM stage (AJCC)				0.022*
I and II	23	10	13	
III and IV	23	3	20	
CRP (mg/l)				0.624
< 10	33	10	23	
≥ 10	13	3	10	
CEA (ng/ml)				0.295
< 5	30	10	20	
≥ 5	16	3	13	
CA199 (U/ml)				
< 37	6	2	4	0.767
≥ 37	40	11	29	
CA125 (U/ml)				0.146
< 35	24	9	15	
≥ 35	22	4	18	
CA242 (U/ml)				0.720
< 20	16	4	12	
≥ 20	30	9	21	
CA211 (ng/ml)				0.299
< 5	34	11	23	
≥ 5	12	2	10	

* *p* < 0.05, ** *p* < 0.01

**Table 3:** Association between circulating miR-1290 expression and clinicopathologic characteristics in 50 colorectal cancer patients

Variable	Total (*n* = 50)	miR-1290 expression		*p* value
		Low (*n* = 23)	High (*n* = 27)	
Age (year)	64.00 ± 1.62	63.26 ± 2.46	64.63 ± 2.19	0.679
Sex				0.057
Male	33	12	21	
Female	17	11	6	
BMI (kg/m^2^)	23.83 (21.33–25.54)	24.24 (20.96–27.64)	23.36 (21.45–25.24)	0.483
Smoke				0.741
Yes	23	10	13	
No	27	13	14	
Alcohol				0.990
Yes	13	6	7	
No	37	17	20	
Hypertension				0.309
Yes	19	7	12	
No	31	16	15	
Diabetes				0.777
Yes	5	2	3	
No	45	21	24	
Location				0.555
colon	24	10	14	
rectum	26	13	13	
Tumor size (cm)	3.50 (3.00–3.05)	3.00 (2.30–4.00)	4.00 (3.00–5.00)	0.037*
Lymphatic invasion				0.019*
Positive	22	6	16	
Negative	28	17	11	
Venous invasion				0.019*
Positive	22	6	16	
Negative	28	17	11	
Distant metastasis				0.041*
Positive	16	4	12	
Negative	34	19	15	
Differentiation				0.025*
Well	1	1	0	
Moderate	39	21	18	
Poor	10	1	9	
TNM stage (AJCC)				0.047*
I and II	25	15	10	
III and IV	25	8	17	
CRP (mg/l)				0.918
< 10	41	19	22	
≥ 10	9	4	5	
CEA (ng/ml)				0.022*
< 5	33	19	14	
≥ 5	17	4	13	
CA199 (U/ml)				0.400
< 37	41	20	21	
≥ 37	9	3	6	
CA125 (U/ml)				0.351
< 35	49	23	26	
≥ 35	1	0	1	
CA242 (U/ml)				0.183
< 20	48	23	25	
≥ 20	2	0	2	
CA211 (ng/ml)				0.650
< 5	47	22	25	
≥ 5	3	1	2	

* *p* < 0.05

**Table 4:** Association between circulating miR-1290 expression and clinicopathologic characteristics in 50 gastric cancer patients.

Variable	Total (*n*=50)	miR-1290 expression		*p* value
		Low (*n*=33)	High (*n*=17)	
Age (year)	63.74±1.62	65.58±1.78	60.18±3.19	0.115
Sex				0.616
Male	26	18	8	
Female	24	15	9	
BMI (kg/m^2^)	22.33 (20.50-25.11)	22.27 (20.38-25.06)	22.48 (20.49-25.50)	0.690
Smoke				0.334
Yes	13	10	3	
No	37	23	14	
Alcohol				0.520
Yes	12	7	5	
No	38	26	12	
Hypertension				0.031*
Yes	12	11	1	
No	38	22	16	
Diabetes				0.134
Yes	4	4	0	
No	46	29	17	
Tumor size (cm)	4.00 (1.93-5.50)	4.50 (2.00-5.45)	4.00 (0.70-7.00)	0.498
Lymphatic invasion				0.261
Positive	29	21	8	
Negative	21	12	9	
Venous invasion				0.370
Positive	25	18	7	
Negative	25	15	10	
Distant metastasis				0.613
Positive	14	10	4	
Negative	36	23	13	
Differentiation				0.526
Well	9	5	4	
Moderate	12	7	5	
Poor	29	21	8	
TNM stage (AJCC)				0.754
I and II	28	19	9	
III and IV	22	14	8	
CRP (mg/l)				0.218
< 10	47	32	15	
≥ 10	3	1	2	
CEA (ng/ml)				0.235
< 5	43	27	16	
≥ 5	7	6	1	
CA199 (U/ml)				0.410
< 37	41	26	15	
≥ 37	9	7	2	
CA125 (U/ml)				0.963
< 35	41	27	14	
≥ 35	9	6	3	
CA242 (U/ml)				0.692
< 20	46	30	16	
≥ 20	4	3	1	
CA211 (ng/ml)				0.486
< 5	45	29	16	
≥ 5	5	4	1	

*p < 0.05

A receiver operating characteristic (ROC) curve analysis was performed to evaluate the potential diagnostic value of the circulating miR-1290 level. The area under the curve (AUC) was 0.8857 (*p* < 0.0001) in patients with PC, with 60.9% sensitivity and 90.0% specificity. The AUC was 0.7852 (*p* < 0.0001) for circulating miR-1290 in patients with CRC, with a sensitivity of 42.0% and specificity of 90.0%. In patients with GC, the AUC of miR-1290 was 0.6576 (*p* < 0.01), with a sensitivity of 26.0% and specificity of 90.0%. Moreover, as shown in Fig. 4g-i, the combination of circulating miR-1290 and traditional biomarkers had higher diagnostic value. In patients with PC, the sensitivity and specificity of miR-1290 combined with CA199 was 80.4 and 90.0%, respectively, with an AUC of 0.9626 (*p* < 0.0001). In patients with CRC, the sensitivity and specificity of miR-1290 combined with CEA was 58.0 and 90.0%, respectively, with an AUC of 0.8348 (*p* < 0.0001). The sensitivity and specificity of miR-1290 combined with CA211 was 38.0 and 90.0% in patients with GC, with an AUC of 0.7788 (*p* < 0.0001). As a result, miR-1290 may represent a potential diagnostic biomarker for gastrointestinal cancer, with different diagnostic efficiencies for different types of tumors.

### miR-1290 is a potential biomarker for gastrointestinal tumor surveillance

**Fig. 5:**
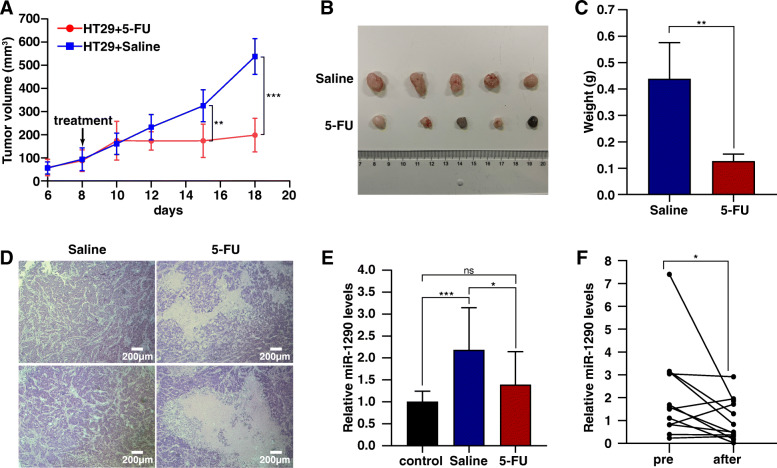
Circulating miR-1290 is a promising biomarker for disease surveillance. **a** The tumor growth curve of different groups. Compared to the saline group, tumor growth in 5-FU-treated group was significantly inhibited. **b** Images of all tumors from nude mouse. Tumors in 5-FU group were significantly smaller than those in saline group. **c** Comparison of tumor weights between saline group and 5-FU group. Tumors in the saline group were heavier than those in the 5-FU group. **d** H&E staining of tumors of saline and 5-FU group. The 5-FU group exhibited a large degree of cell apoptosis and necrosis. **e** The fluctuation of circulating miR-1290 during disease progression and drug treatment in the xenograft mouse model. **f** Changes of serum miR-1290 levels in CRC patients after surgery were determined by RT-qPCR (*n* = 10). * *p* < 0.05, ** *p* < 0.01, *** *p* < 0.001

A subcutaneous xenograft model of CRC in nude mice was constructed to evaluate the fluctuation in the circulating miR-1290 level during disease progression and drug treatment. Eight days after cell inoculation, the tumors grew to about 100 mm^3^ and were treated with 5-FU or the same amount of saline, respectively. As shown in Fig. 5a, the tumor growths in 5-FU-treated group were significantly inhibited. Tumors of the saline group had larger volumes and weights than those of the 5-FU group (*p* < 0.01) (Fig. 5b-c). The circulating miR-1290 level increased significantly after tumor formation (*p* < 0.001) (Fig. 5e). After treatment with 5-FU, the tumor tissue structure was destroyed and tumor cells were largely necrotic (Fig. 5d), with a remarkable reduction in the circulating miR-1290 level (*p* < 0.05) (Fig. 5e). Furthermore, we explored the changes in miR-1290 expression in 10 patients with CRC after surgery. As shown in Fig. 5f, circulating miR-1290 expression decreased substantially after surgery (*p* < 0.05). Therefore, circulating miR-1290 is a promising biomarker for gastrointestinal tumor surveillance.

## Discussion

In this study, we identified circulating miR-1290 as an effective biomarker for the early diagnosis and monitoring of gastrointestinal tumors. Based on previous studies, we further confirmed that miR-1290 was up-regulated in tumor tissues and serum from patients with CRC. High miR-1290 expression was detected in various gastrointestinal cancer cell lines (PC, CRC, and GC), and miR-1290 was released into the culture supernatant. The diagnostic efficiency of the circulating miR-1290 level was evaluated in patients with PC, CRC, and GC by establishing a reliable detection method for cell-free miRNAs. The results indicated that circulating miR-1290 is a potential biomarker for gastrointestinal tumors and that high miR-1290 expression is significantly correlated with disease progression.

Valuable early screening and monitoring indicators can improve patient prognosis and reduce the risk of over-treatment resulting from overdiagnosis [3]. Compared with cellular RNA species, circulating miRNAs are highly stable during long-term storage and extreme conditions, including boiling, multiple freeze-thaw cycles, RNase treatment, and high or low pH [8], making the evaluation of circulating miRNAs an essential part of liquid biopsy [9]. As shown in our previous study, miR-1290 is up-regulated in CRC tissue and plays a crucial role in cancer progression [10]. According to previous studies, miR-1290 exerts a tumor-promoting effect and is involved in the occurrence and development of various tumors, such as CRC [11] and esophageal cancer [12]. In gastric cancer, miR-1290 was highly expressed and promoted cell proliferation and metastasis [13], it might be a potential exosome-based biomarker [14]. Ang Li et al. found that elevated serum miR-1290 accurately distinguished low stage pancreatic patients with healthy controls [15]. Serum miR-1290 might be a promising biomarker for diagnosis of pancreatic cancer [16]. Furthermore, miRNA sequencing indicated that miR-1290 is expressed at high levels in the serum of patients with CRC, suggesting that it might represent a potential gastrointestinal tumor marker.

Cell-free miRNA detection technology is maturing, but many problems still remain to be solved before its clinical application. For example, a high extraction efficiency should be guaranteed due to the relatively low concentration of circulating miRNA, the integrity of miRNA and the stability of detection. Particularly, when a single miRNA is chosen as a clinical biomarker, verification of pre-analysis steps affecting miRNA quantification is critically important. Although highly up-regulated or down-regulated miRNAs have advantages as biomarkers, the clinical value of most markers depends on solving the fore-mentioned problems. However, many researches have not performed methodological verification [17]. Considering the difference between miRNA expression and the molecular mechanism, a detection method applicable to one study is not necessarily suitable for another. Consequently, the detection methodology (reliability, repeatability, and accuracy) for a specific miRNA must be verified and improved [18].

We selected the extraction kit with the highest extraction efficiency (miRNeasy Serum/Plasma Advanced Kit) as the main detection method for subsequent experiments among a variety of kits to ensure a high extraction efficiency. The main extraction principles of this kit are protein precipitation and silica technology, which reduce the inhibitory effects of interfering substances in the blood on the reverse transcription and PCR procedure. Then, we established a standard curve to analyze the recovery rate, which was within the acceptable error range and not affected by bilirubin, rheumatoid factor, or triglyceride in the serum. Thus, our method is reliable. In addition, standard normalization methods for circulating miRNA analyses are still lacking [19]. Notably, miR-16-5p is a widely used endogenous control for circulating miRNA normalization [15]. As shown in previous studies, miR-16-5p is up-regulated in patients with numerous diseases, such as rheumatoid arthritis [20], breast cancer [21], and esophageal cancer [22], and serves as a potential disease marker. In our study, we also confirmed that miR-16-5p is overexpressed in the serum of patients with breast cancer and rheumatoid arthritis, the expression level of circulating miR-16-5p fluctuates with changes in disease activity in patients with gastrointestinal tumors, and normalization with miR-16-5p might decrease variability, as discovered in previous research [7]. Therefore, an external control (cel-miR-39) is preferred.

In the present study, the expression level of miR-1290 in the plasma was higher than that in the serum from the same individual. Contamination of blood cells and PCR amplification processes are the main factors affecting the results [23]. Circulating miRNAs are contaminated with miRNAs from blood cells during coagulation or the separation process of plasma, and hemolysis leads to the release of miRNA from red blood cells [24]. In addition, the anticoagulants and blood stabilizers used during plasma collection also affect the quantitation of circulating miRNAs [25]. The plasma collection process affects the recovery rate and accuracy, regardless of the miRNA expression level. Notably, high RNA concentrations can reduce the interference of plasma components in PCR quantification, indicating that, when cel-miR-39 is used as an exogenous control, the amount added should be maintained at an appropriate and consistent level. Catherine Foye et al also discovered that the results for the detection of endogenous and exogenous controls varied in the serum and plasma, with higher expression levels in plasma [26]. Therefore, the appropriate samples and standardized blood sample processing procedures must be selected to reduce the detection errors in clinical analysis.

Many commonly used biomarkers for malignant tumors of the digestive tract have a broad spectrum. We compared the expression level and diagnostic efficiency of miR-1290 in patients with PC, CRC, and GC to comprehensively understand the diagnostic value of miR-1290 as a biomarker. Significantly higher miR-1290 expression was observed in patients with PC, CRC, and GC than in control, with the highest diagnostic efficiency observed in patients with PC. Previous studies have confirmed the diagnostic value of circulating miR-1290 levels in patients with gastrointestinal tumors, for example, PC [15, 27], CRC [28, 29], and esophageal squamous-cell carcinoma [30]. However, these studies were all conducted in patients with one specific tumor and lacked an evaluation of the role of miR-1290 in different types of tumors. In our study, high miR-1290 expression in the serum was associated with tumor size, lymphatic invasion, venous invasion, distant metastasis, differentiation, and TNM stage (AJCC) in patients with PC and CRC, while this relationship was not observed in patients with GC. The circulating miR-1290 level might be a potential marker for invasiveness and disease progression in patients with PC and CRC, while the correlation with the aggressiveness of GC was not strong. Patients with CRC with high miR-1290 expression levels also had relatively high CEA levels, while no association between the level of miR-1290 expression and that of other tumor markers was observed. In patients with PC, correlations were not observed between the level of miR-1290 expression and that of other common biomarkers. Based on these results, the serum/plasm miR-1290 level represents an independent biomarker with great value as a reference in diagnosing tumors and determining a patient’s condition. We also explored the role of miR-1290 in disease monitoring. Based on the combination of data from clinical patients and the xenograft mouse models, we concluded that the circulating miR-1290 level fluctuated during tumor development and treatment, playing an important role in the real-time monitoring of tumor progression. Although similar studies have been conducted in the past, our study focused on the establishment and verification of the methodology and compared the efficiency of miR-1290 in diagnosing various gastrointestinal tumors. In addition, data from clinical samples and an animal model were combined to ensure the reliability of the results and the clinical significance of the research conclusions.

This study has several limitations. First, the sample size was small. A large sample size from a multicenter study is needed to further confirm the conclusions. Second, it would be more convincing to evaluate the role of circulating miR-1290 in other gastrointestinal tumors. Third, we have not explored the correlation between miR-1290 expression and patient prognosis. We plan to conduct a long-term clinical follow-up study to determine whether the circulating miR-1290 level can serve as a prognostic biomarker. In addition, previous studies have suggested that miR-1290 is involved in the regulation of chemotherapy resistance in a variety of tumors [31]. Ling Ye et al discovered that tissue miR-1290 expression levels were positively correlated with the dMMR status and predicted the prognosis of patients with CRC receiving 5-FU treatment [32]. The circulating miR-1290 level was also a potential biomarker for the response of patients with advanced oral squamous cell cancer to 5-FU-based chemoradiotherapy [33]. Therefore, the relationship between circulating miR-1290 expression and chemotherapy resistance in patients with gastrointestinal tumors is worthy further study. Furthermore, in-depth studies on the possible molecular mechanism are lacking. The ability of cancer cells to interconvert between epithelial and mesenchymal states was closely related to tumor progression [34–36]. The correlation between miR-1290 and epithelial-mesenchymal plasticity (EMP) remains to be discussed [37–39]. Finally, the combined diagnosis using multiple different markers also have great research value.

## Conclusions

We have established a reliable method for the detection of circulating miRNAs, and circulating miR-1290 is a potential biomarker for the diagnosis and monitoring of human gastrointestinal tumors.

## Supplementary Information

**Additional file 1 **: **Table S1.** Highly expressed serum miRNAs in patients with CRC compared to normal controls. **Table S2.** Different RNA extraction technologies utilized in this study. **Table S3.** Mean C_q_ values and SD of circulating miR-1290 measured by RT-qPCR in 20 healthy individuals with different RNA extraction methods. **Table S4.** Recovery efficiency and the influence of common interference factors. **Fig. S1.** Functional enrichment analysis of target genes and phylogenetic analysis of miRNAs. (a-b) GO term enrichment analysis and KEGG pathway analysis of target genes. Top three terms with FDR < 0.01. (c) The phylogenetic tree of miRNAs shown in Table S1 based on the p distance and the average method. **Fig. S2.** The interaction regulatory network of the selected upregulated miRNAs and downregulated target mRNAs. The red circle represents miRNA, and the blue rectangle represents the target genes.

## Data Availability

The datasets generated and/or analyzed during the current study are available in the Sequence Read Archive (SRA) repository (https://www.ncbi.nlm.nih.gov/Traces/study/?acc=PRJNA756902). Accession number: PRJNA756902.

## Abbreviations

CRC: Colorectal cancer
PC: Pancreatic cancer
GC: Gastric cancer
ROC curve: Receiver operating characteristic curve
AUC: Area under the curve
