# Supplementary material for: Circulating MiR-1290 as a potential diagnostic and disease monitoring biomarker of human gastrointestinal tumors
Source: BMC Cancer. 2021 Sep 3;21:989. doi: 10.1186/s12885-021-08729-0 (PMC8417985; doi:10.1186/s12885-021-08729-0)
Supplement: Supplementary file 1 — Additional file 1 : Table S1. Highly expressed serum miRNAs in patients with CRC compared to normal controls. Table S2. Different RNA extraction technologies utilized in this study. Table S3. Mean Cq values and SD of circulating miR-1290 measured by RT-qPCR in 20 healthy individuals with different RNA extraction methods. Table S4. Recovery efficiency and the influence of common interference factors. Fig. S1. Functional enrichment analysis of target genes and phylogenetic analysis of miRNAs. (a-b) GO term enrichment analysis and KEGG pathway analysis of target genes. Top three terms with FDR < 0.01. (c) The phylogenetic tree of miRNAs shown in Table S1 based on the p distance and the average method. Fig. S2. The interaction regulatory network of the selected upregulated miRNAs and downregulated target mRNAs. The red circle represents miRNA, and the blue rectangle represents the target genes. [file 12885_2021_8729_MOESM1_ESM.zip › supplemental material_V2R6.docx]

**Table S1. Highly expressed serum miRNAs in patients with CRC compared to normal controls.**

| Name | MiRbase accession number | Sequence | Regulation | Fold change | p value |
| --- | --- | --- | --- | --- | --- |
| hsa-miR-203a-3p | MIMAT0000264 | GUGAAAUGUUUAGGACCACUAG | up | 45.27 | 0.00E+00 |
| hsa-miR-122-5p | MIMAT0000421 | UGGAGUGUGACAAUGGUGUUUG | up | 12.25 | 0.00E+00 |
| hsa-miR-192-5p | MIMAT0000222 | CUGACCUAUGAAUUGACAGCC | up | 10.39 | 0.00E+00 |
| hsa-miR-150-5p | MIMAT0000451 | UCUCCCAACCCUUGUACCAGUG | up | 9.55 | 0.00E+00 |
| hsa-miR-194-5p | MIMAT0000460 | UGUAACAGCAACUCCAUGUGGA | up | 9.50 | 0.00E+00 |
| hsa-miR-375-3p | MIMAT0000728 | UUUGUUCGUUCGGCUCGCGUGA | up | 8.50 | 0.00E+00 |
| hsa-miR-483-5p | MIMAT0004761 | AAGACGGGAGGAAAGAAGGGAG | up | 6.22 | 0.00E+00 |
| hsa-miR-99a-5p | MIMAT0000097 | AACCCGUAGAUCCGAUCUUGUG | up | 6.16 | 0.00E+00 |
| hsa-miR-100-5p | MIMAT0000098 | AACCCGUAGAUCCGAACUUGUG | up | 5.35 | 0.00E+00 |
| hsa-miR-146b-5p | MIMAT0002809 | UGAGAACUGAAUUCCAUAGGCUG | up | 4.51 | 0.00E+00 |
| hsa-miR-1290 | MIMAT0005880 | UGGAUUUUUGGAUCAGGGA | up | 4.50 | 0.00E+00 |
| hsa-miR-125b-5p | MIMAT0000423 | UCCCUGAGACCCUAACUUGUGA | up | 4.30 | 0.00E+00 |
| hsa-miR-182-5p | MIMAT0000259 | UUUGGCAAUGGUAGAACUCACACU | up | 3.69 | 0.00E+00 |
| hsa-miR-23b-3p | MIMAT0000418 | AUCACAUUGCCAGGGAUUACCAC | up | 3.30 | 0.00E+00 |
| hsa-miR-10a-5p | MIMAT0000253 | UACCCUGUAGAUCCGAAUUUGUG | up | 3.21 | 0.00E+00 |
| hsa-miR-96-5p | MIMAT0000095 | UUUGGCACUAGCACAUUUUUGCU | up | 3.10 | 0.00E+00 |

**Table S2. Different RNA extraction technologies utilized in this study.**

| Manufacturer | RNA extraction kits | Principle of extraction |
| --- | --- | --- |
| QIAGEN | miRNeasy Serum/Plasma Kit | Phenol/guanidine-chloroform + Silica technology |
| QIAGEN | miRNeasy Serum/Plasma Advanced Kit | Protein precipitation + Silica technology |
| QIAGEN | QIAamp Circulating Nucleic Acid Kit | Proteinase + Silica technology |
| Invitrogen | TRIzol LS Reagent | Phenol/guanidine-chloroform + precipitation |
| Invitrogen | TRIzol Reagent | Phenol/guanidine-chloroform + precipitation |

**Table S3. Mean C_q_ values and SD of circulating miR-1290 measured by RT-qPCR in 20 healthy individuals with different RNA extraction methods.**

|  |  |  | Normalized to | | | | |  |
| --- | --- | --- | --- | --- | --- | --- | --- | --- |
|  |  |  | cel-miR-39 | | miR-16-5p | | |  |
| Methods | Mean raw Cq | SD | Cq | SD | | Cq | SD | |
| miRNeasy Serum/Plasma Kit | 30.33 | 0.90 | 30.79 | 0.34 | | 30.68 | 0.54 | |
| miRNeasy Serum/Plasma Advanced Kit | 27.78 | 1.12 | 27.81 | 2.07 | | 27.67 | 1.50 | |
| QIAamp Circulating Nucleic Acid Kit | 33.11 | 0.30 | 33.37 | 0.43 | | 32.98 | 1.05 | |
| TRIzol LS Reagent | 30.59 | 1.66 | 29.75 | 1.06 | | 30.71 | 0.76 | |
| TRIzol Reagent | 32.06 | 1.24 | 32.16 | 1.41 | | 32.36 | 1.07 | |

**Table S4. Recovery efficiency and the influence of common interference factors.**

| Interference factor | Sample | Copies of standards | The recovery amounts | Recovery rate (%) |
| --- | --- | --- | --- | --- |
| - | control | - | 58479603.74 | - |
| - | control+ standards | 1204000000 | 1115751731 | 87.8 |
| - | control+ standards | 120400000 | 198074269.5 | 116 |
| - | control+ standards | 12040000 | 70998961.56 | 103 |
| bilirubin |  |  |  |  |
|  | control | - | 374765885.9 |  |
|  | control+ standards | 1204000000 | 1813957564 | 119 |
| Triglyceride |  |  |  |  |
|  | control | - | 87034924.88 |  |
|  | control+ standards | 1204000000 | 1207162807 | 93.0 |
| rheumatoid factor |  |  |  |  |
|  | control | - | 83035384.09 |  |
|  | control+ standards | 1204000000 | 1369771036 | 107 |

**Figure legend**

**Fig S1.** **Functional enrichment analysis of target genes and** **phylogenetic analysis of miRNAs.** (a-b) GO term enrichment analysis and KEGG pathway analysis of target genes. Top three terms with FDR<0.01. (c) The phylogenetic tree of miRNAs shown in Table S1 based on the p distance and the average method.

**Fig S2. The interaction regulatory network of the selected upregulated miRNAs and downregulated target mRNAs.** The red circle represents miRNA, and the blue rectangle represents the target genes.
