# Supplementary figures and images for: Circulating MiR-1290 as a potential diagnostic and disease monitoring biomarker of human gastrointestinal tumors
Source: BMC Cancer. 2021 Sep 3;21:989. doi: 10.1186/s12885-021-08729-0 (PMC8417985; doi:10.1186/s12885-021-08729-0)

**A**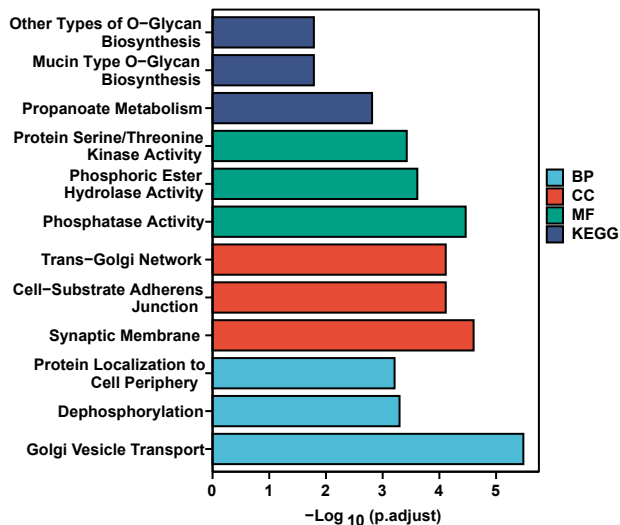**B**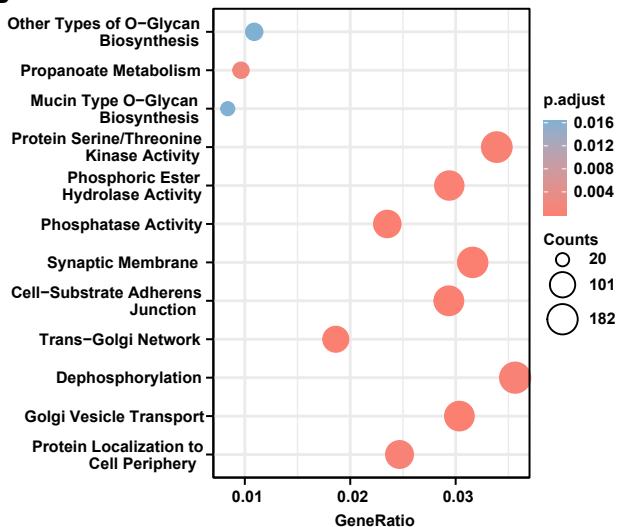**C**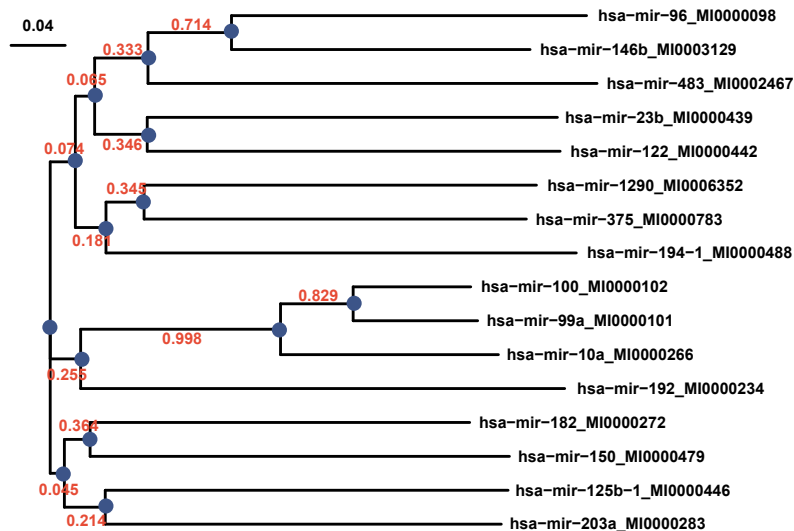

Supplement: Supplementary file 1 — Additional file 1 : Table S1. Highly expressed serum miRNAs in patients with CRC compared to normal controls. Table S2. Different RNA extraction technologies utilized in this study. Table S3. Mean Cq values and SD of circulating miR-1290 measured by RT-qPCR in 20 healthy individuals with different RNA extraction methods. Table S4. Recovery efficiency and the influence of common interference factors. Fig. S1. Functional enrichment analysis of target genes and phylogenetic analysis of miRNAs. (a-b) GO term enrichment analysis and KEGG pathway analysis of target genes. Top three terms with FDR < 0.01. (c) The phylogenetic tree of miRNAs shown in Table S1 based on the p distance and the average method. Fig. S2. The interaction regulatory network of the selected upregulated miRNAs and downregulated target mRNAs. The red circle represents miRNA, and the blue rectangle represents the target genes. [file 12885_2021_8729_MOESM1_ESM.zip › Figure S1R6.pdf]

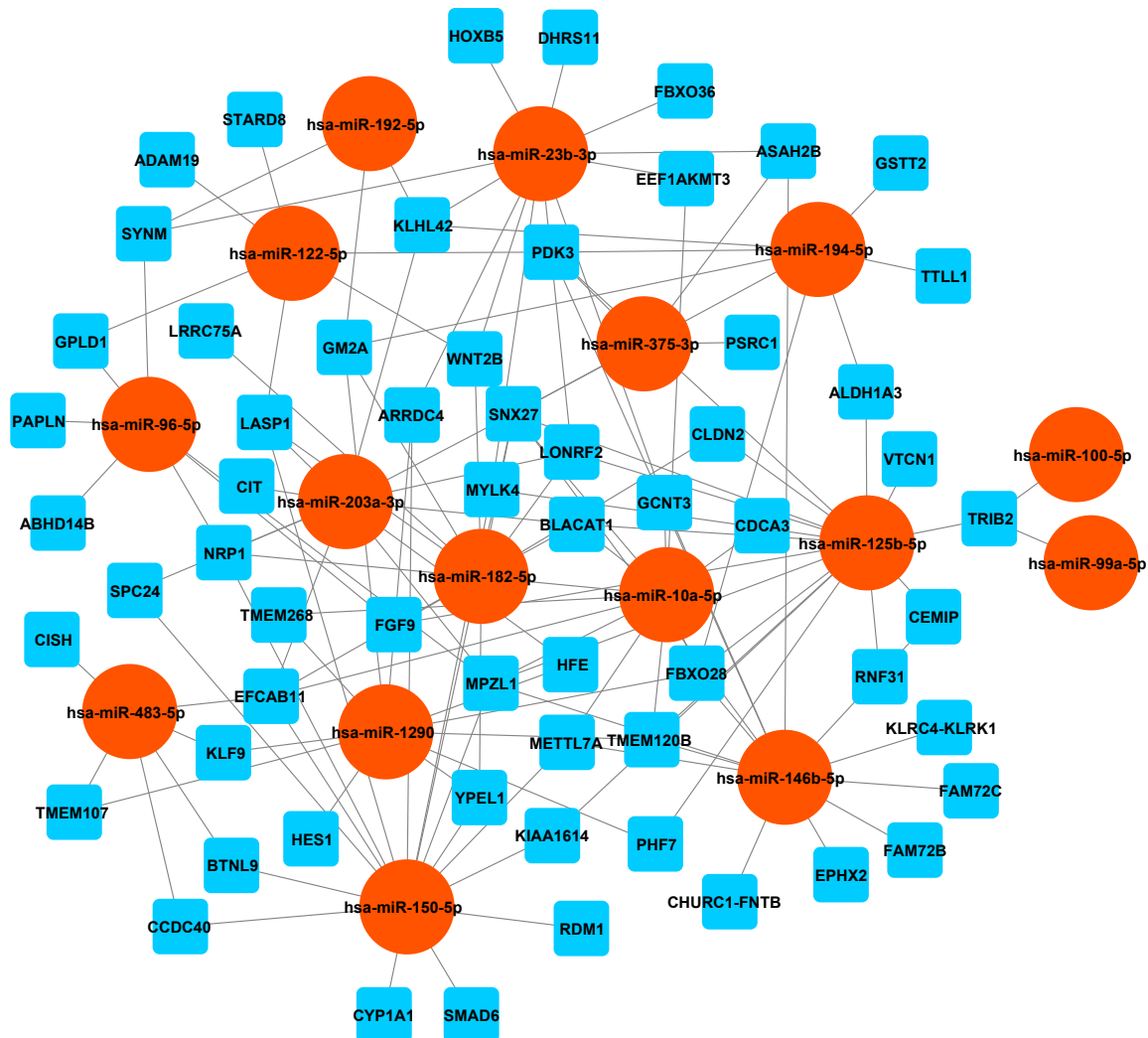

Supplement: Supplementary file 1 — Additional file 1 : Table S1. Highly expressed serum miRNAs in patients with CRC compared to normal controls. Table S2. Different RNA extraction technologies utilized in this study. Table S3. Mean Cq values and SD of circulating miR-1290 measured by RT-qPCR in 20 healthy individuals with different RNA extraction methods. Table S4. Recovery efficiency and the influence of common interference factors. Fig. S1. Functional enrichment analysis of target genes and phylogenetic analysis of miRNAs. (a-b) GO term enrichment analysis and KEGG pathway analysis of target genes. Top three terms with FDR < 0.01. (c) The phylogenetic tree of miRNAs shown in Table S1 based on the p distance and the average method. Fig. S2. The interaction regulatory network of the selected upregulated miRNAs and downregulated target mRNAs. The red circle represents miRNA, and the blue rectangle represents the target genes. [file 12885_2021_8729_MOESM1_ESM.zip › Figure S2R6.pdf]
